# Supplementary material for: Nuclear pores safeguard the integrity of the nuclear envelope
Source: Nat Cell Biol. 2025 Apr 9;27(5):762–75. doi: 10.1038/s41556-025-01648-3 (PMC12081302; doi:10.1038/s41556-025-01648-3)
Supplement: Supplementary file 1 — Supplementary Table 1: Statistics for data acquisition and subtomogram averaging. [file 41556_2025_1648_MOESM1_ESM.pdf]

nature cell biology

Supplementary information https://doi.org/10.1038/s41556-025-01648-3

Nuclear pores safeguard the integrity of the nuclear envelope

In the format provided by the authors and unedited

Dataset	Wild-type mES cells			Wild-type neural progenitor cells			*Nup133*$^{-/-}$ mES cells									*Nup133*$^{-/-}$ neural progenitor cells			Monocyte-derived macrophages
Microscope	Titan Krios G2			Titan Krios G2			Titan Krios G2									Titan Krios G2			Titan Krios G4
Voltage (kV)	300			300			300									300			300
Camera	Gatan K3			Gatan K3			Gatan K3									Gatan K3			TFS Falcon4
Magnification	33000			33000			33000									33000			53000
Pixel size (Å/pixel)	2.682			2.682			2.682									2.682			2.414
Targeted total electron exposure ($e^-/Å^2$)	~150			~150			~150									~150			~135
Targeted defocus range (μm)	-2.5 - -4.5			-2.5 - -4.5			-2.5 - -4.5									-2.5 - -4.5			-2.0 - -4.0
Automation software	SerialEM			SerialEM			SerialEM									SerialEM			SerialEM
Tilt-series collected	260			176			280									237			75
Tomograms used for NPC diameter analysis	161			96			164									144			-
Tomograms used for STA	109			64			123									104			4*
initial # of NPC	447			180			411									193			
Symmetry	8-fold			8-fold			7-fold			8-fold			9-fold			8-fold			
Selected # of NPC	447			180			34			323			32			157			
Map type	CR	IR**	NR	CR	IR**	NR	CR	IR	NR	CR	IR**	NR	CR	IR	NR	CR	IR**	NR	
Final # of particles	1450	1452 (3528)	1451	851	851 (1338)	851	238	238	238	970	970 (2510)	970	288	288	288	894	894 (1073)	894	
Resolution (Å)***	28.7	28.5 (26.5)	30.4	31.8	30.7 (30.2)	31.0	-	48.2	-	31.1	30.1 (28.8)	33.4	48.3	50.3	-	34.2	30.2 (29.9)	33.8	

* Number of tomograms used for the template matching analysis of the over-stretchend NPCs

** Numbers in brackets correspond to the dataset used for STA-based NPC diameter measurement.

*** Resolution calculated using FSC = 0.143 threshold

Supplementary Table 1
