# Supplementary material for: Nuclear pores safeguard the integrity of the nuclear envelope
Source: Nat Cell Biol. 2025 Apr 9;27(5):762–75. doi: 10.1038/s41556-025-01648-3 (PMC12081302; doi:10.1038/s41556-025-01648-3)
Supplement: Supplementary file 2 — Reporting Summary [file 41556_2025_1648_MOESM2_ESM.pdf]

nature portfolio

Corresponding author(s): Martin Beck

Last updated by author(s): Feb 3, 2025

Reporting Summary

Nature Portfolio wishes to improve the reproducibility of the work that we publish. This form provides structure for consistency and transparency in reporting. For further information on Nature Portfolio policies, see our Editorial Policies and the Editorial Policy Checklist.

Statistics

For all statistical analyses, confirm that the following items are present in the figure legend, table legend, main text, or Methods section.

n/a	Confirmed	
☐	☒	The exact sample size (n) for each experimental group/condition, given as a discrete number and unit of measurement
☐	☒	A statement on whether measurements were taken from distinct samples or whether the same sample was measured repeatedly
☐	☒	The statistical test(s) used AND whether they are one- or two-sided<br>*Only common tests should be described solely by name; describe more complex techniques in the Methods section.*
☒	☐	A description of all covariates tested
☒	☐	A description of any assumptions or corrections, such as tests of normality and adjustment for multiple comparisons
☐	☒	A full description of the statistical parameters including central tendency (e.g. means) or other basic estimates (e.g. regression coefficient) AND variation (e.g. standard deviation) or associated estimates of uncertainty (e.g. confidence intervals)
☐	☒	For null hypothesis testing, the test statistic (e.g. F, t, r) with confidence intervals, effect sizes, degrees of freedom and P value noted<br>*Give P values as exact values whenever suitable.*
☒	☐	For Bayesian analysis, information on the choice of priors and Markov chain Monte Carlo settings
☒	☐	For hierarchical and complex designs, identification of the appropriate level for tests and full reporting of outcomes
☒	☐	Estimates of effect sizes (e.g. Cohen's d, Pearson's r), indicating how they were calculated

Our web collection on statistics for biologists contains articles on many of the points above.

Software and code

Policy information about availability of computer code

Data collection	SerialEM(3.8.5)
Data analysis	gctf(1.06), IMOD(version 4.10.9 and 4.11.5), AreTomo (1.33), novaCTF (https://github.com/turonova/novaCTF), novaSTA (https://github.com/turonova/novaSTA), Dynamo(1.1.532), Relion(version 3.1), STOPGAP(version 0.7.1, https://github.com/wan-lab-vanderbilt/STOPGAP), MATLAB R2019b/R2022b, ChimeraX (1.5), Membrain-seg (https://github.com/teamtomo/membrain-seg), Amira Software (Thermo Scientific, ver 2023.2), ArtiaX, napari, FiJi (ImagJ version 1.54f), GraphPad Prism 9

For manuscripts utilizing custom algorithms or software that are central to the research but not yet described in published literature, software must be made available to editors and reviewers. We strongly encourage code deposition in a community repository (e.g. GitHub). See the Nature Portfolio guidelines for submitting code & software for further information.

Data

Policy information about availability of data

All manuscripts must include a data availability statement. This statement should provide the following information, where applicable:

- Accession codes, unique identifiers, or web links for publicly available datasets
- A description of any restrictions on data availability
- For clinical datasets or third party data, please ensure that the statement adheres to our policy

Our final data availability statement says "The subtomogram averages described in this study are deposited in the Electron Microscopy Data Bank (EMDB) with the

accession codes EMD-52153, EMD-52154, EMD-52155, EMD-52156, EMD-52157, EMD-52158, EMD-52159, EMD-52160, EMD-52161, EMD-52162, EMD-52163, and EMD-52164. The composite maps of the C8-symmetrized whole NPCs are deposited as supplementary maps of EMD-52153, EMD-52156, EMD-52159, and EMD-52162. The raw tilt series are deposited in the Electron Microscopy Public Image Archive (EMPIAR) with the accession codes EMPIAR-12457, EMPIAR-12460, EMPIAR-12461, EMPIAR-12462, and EMPIAR-12463. Light microscopy images used for quantitative analyses are deposited in the BioImage Archive with the accession codes S-BIAD1493, S-BIAD1494, and S-BIAD1495. Source data have been provided in Source Data. All other data supporting the findings of this study are available from the corresponding author on reasonable request". All the relevant data have been deposited to respective databases.

Research involving human participants, their data, or biological material

Policy information about studies with human participants or human data. See also policy information about sex, gender (identity/presentation), and sexual orientation and race, ethnicity and racism.

Reporting on sex and gender	This study does not involve human research participants. The cryo-ET dataset on primary human macrophages we analyzed was acquired as part of another study (doi:10.1016/j.cell.2024.12.008), where the sample preparation is described in detail. In brief, Monocyte-derived macrophages (MDMs) were obtained from human peripheral blood mononuclear cells (PBMC) isolated from buffy coats of anonymous blood donors at the Heidelberg University Hospital Blood Bank according to the regulations of the local ethics committee.
Reporting on race, ethnicity, or other socially relevant groupings	not applicable, see above
Population characteristics	not applicable, see above
Recruitment	not applicable, see above
Ethics oversight	Local ethics committee at the Heidelberg University Hospital

Note that full information on the approval of the study protocol must also be provided in the manuscript.

Field-specific reporting

Please select the one below that is the best fit for your research. If you are not sure, read the appropriate sections before making your selection.

☒ Life sciences ☐ Behavioural & social sciences ☐ Ecological, evolutionary & environmental sciences

For a reference copy of the document with all sections, see nature.com/documents/nr-reporting-summary-flat.pdf

Life sciences study design

All studies must disclose on these points even when the disclosure is negative.

Sample size	We did not pre-determine the sample size when the study was being designed. We prepared three to six different grids for each sample and acquired the maximum amount of cryo-ET data within the available machine time. For the other experiments, we determined the sample size based on similar experiments performed in previous literatures and the available time for data acquisition.
Data exclusions	None.
Replication	The Cryo-ET data of the wild-type and Nup133KO mES cells were acquired from six EM grids prepared in three and two independent plunge freezing sessions, respectively. The cryo-ET data of the neural progenitor samples were acquired from three EM grids prepared in three independent plunge freezing sessions. All the data from different data acquisition sessions show consistent results. Differentiation experiments were repeated seven times and consistent results were obtained. Immunofluorescence staining experiments were performed at least three times, and all the attempts were successful.
Randomization	We did not use randomization in our study. For the experiments involving mES cells, the wild-type and Nup133KO cell lines were always compared under the same conditions throughout the study, and all the experiments were conducted for both cell lines using the same type of dishes and same batch of reagents to minimize experimental biases. The data analyses were also conducted in the same way between two cell lines.
Blinding	We did not use blinding in our study. For both the EM and light microscopy imaging experiments, blinding was not practical, because Nup133KO-specific phenotypes were in many cases readily visible in the datasets. For structural analyses, blinding is not applicable due to the necessity of combining the data from the same cell line.

Reporting for specific materials, systems and methods

We require information from authors about some types of materials, experimental systems and methods used in many studies. Here, indicate whether each material, system or method listed is relevant to your study. If you are not sure if a list item applies to your research, read the appropriate section before selecting a response.

Materials & experimental systems

n/a	Involved in the study
☐	☒ Antibodies
☐	☒ Eukaryotic cell lines
☒	☐ Palaeontology and archaeology
☒	☐ Animals and other organisms
☒	☐ Clinical data
☒	☐ Dual use research of concern
☒	☐ Plants

Methods

n/a	Involved in the study
☒	☐ ChIP-seq
☒	☐ Flow cytometry
☒	☐ MRI-based neuroimaging

Antibodies

Antibodies used

mouse anti-Pax6 (Developmental Studies Hybridoma Bank, RRID AB_528427, dilution 1:12), mouse anti-Oct3/4 (Santa Cruz Biotechnology, sc-5279, dilution 1:200), mouse anti-γ-H2AX (Merck, 05-636, dilution 1:1000), rabbit anti-53BP1 (Novus Biologicals, NB100-304SS, dilution 1:1000), rabbit anti-YAP (Cell Signaling Technology, #14074, dilution 1:100), goat anti-mouse Alexa Fluor Plus 488-conjugated antibody (Thermo Fisher Scientific, A32723, dilution 1:2000), goat anti-rabbit Alexa Fluor 594-conjugated antibody (Thermo Fisher Scientific, A11012, dilution 1:2000)

Validation

All the antibodies were directly purchased from the supplier. All the primary antibodies have been used multiple times in previous literatures, and were validated for species reactivity and application by each supplier with following validation information;
Anti-Pax6 (DSHB, AB_528427):
Reacts to amphibian, avian, fish, human, lizard, mouse, opossum, planaria, rat, turtle, zebrafish; Suitable for ChIP, FACS, FFPE, Function Blocking, Gel Supershift, IF, IHC, IP, WB
Anti-Oct3/4 (Santa Cruz, sc-5279):
Reacts to mouse, rat and human; Suitable for WB, IP, IF, IHC(P), FCM and ELISA
Anti-γ-H2AX (Merck, 05-636):
Reacts to vertebrates; Suitable for ICC, IF, WB, ChIP, IHC
Anti-53BP1 (Novus Biologicals, NB100-304SS):
Reacts to human, mouse, rat, bovine, canine, fish, goat, primate, porcine, feline, rabbit, sheep; Suitable for WB, ChIP, Flow, Func, ICC/IF, IHC, IP, WB, ChIP
Anti-YAP (Cell Signaling Technology, #14074):
Reacts to human, mouse, rat, hamster, monkey; Suitable for WB, IP, IHC, IF, FCM, ChIP, CUT & RUN

Eukaryotic cell lines

Policy information about cell lines and Sex and Gender in Research

Cell line source(s)

Nup133-knock out mES cell line was obtained from HM1 mES cell line. It was originally established and validated in Valérie Doye group at the Institut Jacques Monod (Souquet et al., Cell Reports, 2018).

Authentication

Cell lines were regularly authenticated based on morphology. To minimize the risk of cross contamination and spontaneous differentiation of mES cells, we only maintained cells in culture for less than 10 passages.

Mycoplasma contamination

Cell lines were tested negative for mycoplasma contamination.

Commonly misidentified lines
(See ICLAC register)

No commonly misidentified cell lines were used in this study.

Plants

Seed stocks

This study does not involve plant samples.

Novel plant genotypes

This study does not involve plant samples.

Authentication

This study does not involve plant samples.
